# Strongly Coupled 𝒫𝒯-Symmetric Models in Holography

**DOI:** 10.3390/e27010013

**Published:** 2024-12-27

**Authors:** Daniel Areán, David Garcia-Fariña, Karl Landsteiner

**Affiliations:** 1 Instituto de Física Teórica UAM/CSIC, Campus de Cantoblanco, c/Nicolás Cabrera 13-15, 28049 Madrid, Spain; david.garciafarinna@estudiante.uam.es (D.G.-F.); karl.landsteiner@csic.es (K.L.); 2 Departamento de Física Teórica, Universidad Autónoma de Madrid, Campus de Cantoblanco, 28049 Madrid, Spain

**Keywords:** non-Hermitian physics, PT symmetry, gauge/gravity duality

## Abstract

Non-Hermitian quantum field theories are a promising tool to study open quantum systems. These theories preserve unitarity if PT symmetry is respected, and in that case, an equivalent Hermitian description exists via the so-called Dyson map. Generically, PT-symmetric non-Hermitian theories can also feature phases where PT symmetry is broken and unitarity is lost. We review the construction of holographic duals to strongly coupled PT-symmetric quantum field theories and the study of their phase diagram. We next focus on spacetime-dependent non-Hermitian couplings: non-Hermitian quenches and lattices. They violate the null energy condition in the gravity dual. The lattices realize phases supporting an imaginary current that breaks PT symmetry spontaneously. Remarkably, these non-Hermitian lattices flow to a PT-symmetric fixed point in the IR.

## 1. Introduction

One of the fundamental axioms of quantum mechanics (QM) is that the Hamiltonian should be Hermitian. This assumption ensures unitary evolution or, equivalently, reality of the eigenvalues of the Hamiltonian. However, when taking unitarity as the true physical restriction, one concludes that it is possible to consistently formulate quantum mechanics with non-Hermitian Hamiltonians [1]. These non-Hermitian theories are instead required to have PT symmetry [1] or, more generally, any antilinear symmetry [2,3]. However, the existence of such symmetry is not a sufficient condition for unitary evolution, as it can be spontaneously broken by the spectrum of the theory. These realizations gave rise to the field of PT-symmetric quantum mechanics (see, for instance, [4,5,6,7,8] for an introduction).

Physically, a non-Hermitian theory is interpreted as involving the influx and outflux of matter. Thus, PT-symmetric theories correspond to the family of non-Hermitian theories where influx and outflux are balanced, ensuring that matter content is conserved. To illustrate these features, we consider the following PT-symmetric two-level Hamiltonian [4]

(1) $$H=\left(\begin{array}{cc} -i\Gamma & g\\ g & i\Gamma \end{array}\right)\;,$$

where Γ and *g* are real. In this theory, PT is given by

(2) $$\mathrm{PT}=\left(\begin{array}{cc} 0 & 1\\ 1 & 0 \end{array}\right)C\;,$$

with C as the complex conjugation operator. Notably here, P and T have the usual interpretation of parity and time reversal.

The eigenstates of the Hamiltonian in Equation (1) are given by

(3) $$\begin{array}{cc} \; & \psi_{+}=\frac{-i\Gamma +\sqrt{g^{2}-\Gamma^{2}}}{g}\left(\begin{array}{c} 1\\ 0 \end{array}\right)+\left(\begin{array}{c} 0\\ 1 \end{array}\right)\;,\;H\psi_{+}=\sqrt{g^{2}-\Gamma^{2}}\;\psi_{+}\;,\\ \; & \psi_{-}=-\frac{i\Gamma +\sqrt{g^{2}-\Gamma^{2}}}{g}\left(\begin{array}{c} 1\\ 0 \end{array}\right)+\left(\begin{array}{c} 0\\ 1 \end{array}\right)\;,\;H\psi_{-}=-\sqrt{g^{2}-\Gamma^{2}}\;\psi_{-}\;. \end{array}$$

When $g^{2}-\Gamma^{2}>0$, the eigenvalues are real and $\psi_{+}$ and $\psi_{-}$ are eigenstates of PT

$$\mathrm{PT}\psi_{\pm }=\frac{i\Gamma \pm \sqrt{g^{2}-\Gamma^{2}}}{g}\psi_{\pm }\;.$$

However, when $g^{2}-\Gamma^{2}<0$, the energies become complex and $\psi_{+}$ and $\psi_{-}$ are no longer eigenstates of PT

$$\mathrm{PT}\psi_{\pm }=\pm i\frac{\Gamma \mp \sqrt{\Gamma^{2}-g^{2}}}{g}\psi_{\mp }\;.$$

Hence, the theory is only PT-symmetric for $g^{2}-\Gamma^{2}>0$, which indeed matches the regime where the energies are real, showcasing the aforementioned relation between unitary evolution and PT symmetry. The value $g^{2}=\Gamma^{2}$, separating the PT-broken and PT-unbroken regimes, is labeled as the exceptional point.

The physical interpretation for this model is the following: Γ encodes the transfer of matter between the exterior and the two levels, and *g* represents a hopping between them. Then, the PT-symmetric regime corresponds to the hopping between levels being faster than the inflow (outflow) of matter from (to) the exterior. Hence, the system is capable of finding equilibrium. Conversely, in the PT-broken regime, the inflow (outflow) is faster than the hopping, and thus, no stationary state is reached.

A particularly important feature of PT-symmetric models is the existence of the Dyson map, a similarity transformation taking a non-Hermitian model with unbroken PT symmetry to a Hermitian theory. Such a map allows PT-symmetric models to be studied in terms of a more conventional Hermitian description. Nonetheless, in many cases, the mapping can be highly non-trivial and give rise to non-local interactions. This alone motivates the study of non-Hermitian theories for their own physical significance. Furthermore, once we remove the constraints of Hermiticity and instead construct PT-symmetric Hamiltonians, it is interesting to study the vacua and the spontaneous breaking of PT as a function of the parameters of the model.

Another relevant aspect of PT-symmetric theories is that energy eigenstates are not orthogonal due to the non-Hermitian nature of the Hamiltonian. This implies that one needs to replace the notion of orthogonality by that of biortogonality [9] such that matrix elements of an operator *A* are given by $\left\langle \tilde{n}|A|m\right\rangle$, where $\left\{|\tilde{n}\rangle \right\}$ is the dual basis, defined such that $\langle \tilde{n}|m\rangle =\delta_{nm}$. Importantly, this implies that the trace of *A* is defined as

(4) $$\mathrm{Tr}\{A\}=\sum_{n}\langle \tilde{n}|A|n\rangle \;,$$

If {|m⟩} is the basis of eigenstates of the Hamiltonian, the dual basis $\left\{|\tilde{n}\rangle \right\}$ are the eigenstates of the conjugate Hamiltonian

(5) $$H|m\rangle =E_{m}|m\rangle \;,\;H^{†}|\tilde{n}\rangle =E_{n}^{*}|\tilde{n}\rangle \;,$$

We note that this formulation is equivalent to the standard one of pseudo-Hermitian quantum mechanics [2,10,11] by noting that in such cases, $\langle \tilde{n}|=\langle n|\gamma$, where γ is the metric operator [8,12].

Since the inception of PT-symmetric quantum mechanics (QM), significant efforts have been made to extend the framework to quantum field theory (QFT) [13]. However, when studying PT-symmetric QFTs, one faces a significant complication absent in QM: most QFTs cannot be solved non-perturbatively. For this reason, many of the studies of PT-symmetric QFTs have been restricted to the weak coupling limit. To name a few, in [14,15,16,17,18,19,20], the authors considered spontaneous symmetry breaking of global and gauge symmetries in PT-symmetric QFTs, and in [21,22], the effects of non-Hermitian spacetime-dependent couplings were explored. However, the perturbative nature of these studies leads us to pose the following question: does the phenomenology change significantly in the strong coupling regime? Gauge/gravity duality (also known as holographic duality or AdS/CFT correspondence) [23,24,25,26,27] offers a promising new avenue to find an answer.

In essence, gauge/gravity duality relates the large-*N*, strong coupling limit of certain (holographic) QFTs to the classical limit of a gravity theory in an asymptotically Anti-de Sitter (AdS) spacetime with an extra dimension, which encodes the renormalization group flow of the QFT. Thus, by solving problems of classical gravity, we can obtain non-perturbative results for a large family of models, which can serve as candidates to probe the phenomenology of strongly coupled PT-symmetric QFTs. In this context, the standard approach, pioneered in [28], is to consider a Hermitian holographic QFT and promote it to a PT-symmetric one by introducing in the action a non-Hermitian coupling to some scalar operator O of the form

(6) $$S_{O}=\int d^{d}x\left(\bar{s}O+s\bar{O}\right)\;,$$

with *s*, $\bar{s}\in R$ couplings (sources) satisfying $\bar{s}\neq s$. By tuning the values of *s* and $\bar{s}$, one can probe different regimes of the theory, finding phases where PT is spontaneously broken and phases where it is not. Remarkably, this kind of construction is quite similar to the one employed in perturbative studies, where non-Hermiticities are also typically introduced through the couplings of the QFT.

The existing literature on PT-symmetric holographic QFTs has been concerned with exploring and generalizing the model introduced in [28]. There, the authors considered a holographic QFT in three spacetime dimensions and introduced the non-Hermiticity through the (constant) source of an operator O of conformal dimension Δ=2, charged under a global U(1) symmetry. In [29], the full phase diagram of this model and its transport properties were derived both at zero and non-zero chemical potential. The authors found phases where PT was spontaneously broken, which were dual to a complex geometry in AdS and, remarkably, they concluded that the Ferrel–Glover–Tinkham (FGT) sum rule for the electric conductivity held for all phases (even those breaking PT). Generalizations to spacetime-dependent sources were studied in [30,31]. The former paper considered non-Hermitian quenches, where the source was time-dependent and interpolated between different non-Hermitian theories. There, the authors found that the null energy condition was violated. It was also emphasized that the Dyson map of the QFT [30] was dual to a gauge transformation in the gravity theory. On the other hand, ref. [31] considered non-Hermitian lattices and junctions where the sources were dependent on one of the spatial coordinates. There, it was observed that a non-Hermitian complex current appeared in the system concentrated around the regions where the derivative of the sources was greater. Interestingly, such current spontaneously breaks PT without the need for complex geometries. However, it was found that PT symmetry is recovered in the infrared (IR) of the theory. The inhomogeneous model [31] flows from a PT-broken UV to a PT-unbroken IR, similar to the results observed in [21] in the perturbative regime.

In this work, we review recent progress in the formulation and study of PT-symmetric holographic QFTs. We focus mostly on the underlying ideas behind the construction of these models and give an outline of the most interesting results. The paper is organized as follows: In Section 2, we give a brief introduction to gauge/gravity duality. In Section 3, we discuss the bottom-up construction of the holographic model introduced in [28]. There, we follow closely the discussion presented in [29,31]. Following this, in Section 4, we present the full phase diagram and the transport properties of the model for constant coupling at zero and non-zero chemical potential. In Section 5, we discuss spacetime-dependent couplings, first in the context of non-Hermitian quenches, and then in the context of non-Hermitian lattices. We briefly recap the main results in Section 6 and conclude with Section 7, where we present a list of future directions and open questions.

## 2. Gauge/Gravity Duality

Here, we provide a very brief heuristic introduction to gauge/gravity duality. We focus on introducing the key ingredients needed for the following sections. For an in-depth review of gauge/gravity duality and its applications, we refer the reader to [25,26,27].

Gauge/gravity duality establishes a relation between a theory of quantum gravity in a (d+1)-dimensional asymptotically AdS spacetime and a non-gravitational QFT in *d* dimensions. Explicitly, it posits that the generating functionals in both theories are equivalent

(7) $$Z_{\mathrm{QFT}}\left[J\right]=Z_{\mathrm{grav}}\left[\phi_{0}=J\right]$$

where the sources *J* in the QFT side are identified with the boundary conditions $\phi_{0}$ on the fields ϕ on the conformal boundary of AdS. Remarkably, gauge/gravity is a strong/weak duality. In the limit of strong coupling and large rank of the gauge group in the QFT side, the gravity dual is weakly coupled and the classical solution dominates

(8) $$Z_{{\mathrm{QFT}}_{d}}\left[J\right]=Z_{{\mathrm{grav}}_{d+1}}\left[\phi_{0}=J\right]\approx e^{S_{\mathrm{grav}}^{\mathrm{on}-\mathrm{shell}}\left[\phi_{0}=J\right]}$$

where $S_{\mathrm{grav}}^{\mathrm{on}-\mathrm{shell}}$ is the gravitational action evaluated on the classical solution. Hence, we reduce computations in a strongly coupled QFT to solving classical equations of motion of gravity with adequate boundary conditions. In particular, correlation functions in the QFT can be computed by taking functional derivatives with respect to $\phi_{0}$

(9) $$\langle O\left(x_{1}\right)\ldots O\left(x_{n})\right\rangle =\frac{\delta^{n}S_{\mathrm{grav}}^{\mathrm{on}-\mathrm{shell}}}{\delta \phi_{0}\left(x_{1}\right)\ldots \;\delta \phi_{0}\left(x_{n}\right)}$$

where O is the operator sourced by *J*, dual to the field ϕ in the gravity side.

To make these ideas more transparent, it is convenient to consider AdS spacetime written in coordinates such that near the conformal boundary (z=0), the metric takes the form

(10) $$ds^{2}\approx \frac{l^{2}}{z^{2}}\left(-dt^{2}+d{\vec{x}}^{2}+dz^{2}\right)$$

where *l* is the AdS scale, which we set to 1 for convenience.

The extra dimension *z* is identified with the energy scale on the QFT. The UV corresponds to z=0, while the dynamics deep in the bulk (z≫0) describe the IR. In fact, one thinks of the gravity theory as a geometrical realization of the renormalization group flow of the QFT. The flow from the UV towards the IR, caused by the addition of a source *J* to some relevant operator, is encoded in the flow from an AdS spacetime at z→0 to a different geometry for z≫0, as a result of the boundary condition $\phi_{0}=J$.

An important property of the duality is that the isometries of the spacetime are identified with symmetries of the QFT. In particular, this implies that the UV QFT has to be invariant under the isometries of AdS, which are those of the conformal group in *d* dimensions. Therefore, the UV QFT is a conformal field theory (CFT). Nonetheless, the isometry group of AdS is typically an isometry of only the z→0 region; while the isometries deep in the bulk differ. This indicates that, generically, in gauge/gravity, we study flows from a CFT in the UV to some IR QFT which need not be conformal. These flows are typically associated with the addition of some scale to the CFT, for instance, by taking non-zero temperature or by sourcing some relevant operator.

The holographic dual to a CFT at finite temperature *T* is given by an AdS black brane with temperature *T*, that is, an asymptotically AdS spacetime with a planar horizon whose Hawking temperature is *T*. On the other hand, sources have to be added through boundary conditions of the fields on the AdS spacetime. To make this explicit, let us consider the asymptotic expansion of a generic bosonic field Φ near the conformal boundary z=0. The equations of motion are second-order in $\partial_{z}$, and hence, the solution is parameterized by two modes, a leading one $\phi_{0}$ and a subleading one $\phi_{1}$:

(11) $$\Phi \approx \phi_{0}\;z^{\Delta_{-}}\left(1+\ldots \right)+\phi_{1}\;z^{\Delta_{+}}\left(1+\ldots \right)$$

The leading mode $\phi_{0}$ is identified with the source *J* of the dual operator O in the QFT. This dual operator has the same tensor structure as the field Φ and has conformal dimension $\Delta_{+}$. Using the relation Equation (9), it can be seen that the vacuum expectation value (VEV) of O is proportional to the subleading mode $\phi_{1}$. The explicit values of $\Delta_{\pm }$ depend on the field Φ; for instance, for a scalar field with mass *m* living in (d+1) dimensions, we have $\Delta_{\pm }=d/2\pm \sqrt{d^{2}/4+m^{2}}$. We note that negative masses are allowed, provided they are above the Breitenlohner–Freedman (BF) bound, which, for the aforementioned scalar, is given by $m^{2}>-d^{2}/4$.

Global symmetries and the corresponding conserved currents in the QFT are dual to gauge symmetries and the associated gauge fields in the gravity side, respectively. For instance, to consider a U(1) global symmetry, we need to study the dynamics of a U(1) gauge field $A_{\mu }$ in an asymptotically AdS spacetime. The gauge field will be dual to the U(1) current operator $J_{\mu }$, and its equations of motion will yield the usual conservation law $\partial_{\mu }J^{\mu }=0$. The leading mode of $A_{\mu }$ is identified with an external gauge field, which, in the dual QFT, would source $J_{\mu }$.

## 3. Bottom-Up Holographic Model

A non-Hermitian holographic model was constructed in [28] and further developed in [29,30,31]. Let us now discuss how to construct it. We follow a bottom-up approach; that is, we first consider the necessary ingredients in the QFT, and then we construct the minimal gravity theory capable of reproducing those features.

From the point of view of the QFT, as stated in the introduction, we want to add the non-Hermicity through the source of an operator O of conformal dimension Δ=2 charged under a global U(1) symmetry. Hence, the action we wish to study is given by

(12) $$S=S_{\mathrm{CFT}}+\int d^{3}x\left(\bar{s}\;O+s\;\bar{O}\right)$$

where $S_{\mathrm{CFT}}$ is the Hermitian action, which we assume to be PT-symmetric; and we have taken the QFT to live in three spacetime dimensions to match the setup of [28].

Defining the action of PT on the coordinates as

(13) $$x=\left(t,x^{1},x^{2}\right)\overset{\mathrm{PT}}{\to }\mathrm{PT}x=\left(-t,-x^{1},x^{2}\right)\;,$$

and taking O to be a scalar under PT

(14) $$O\left(x\right)\overset{\mathrm{PT}}{\to }O\left(\mathrm{PT}x\right)\;,\;\bar{O}\left(x\right)\overset{\mathrm{PT}}{\to }\bar{O}\left(\mathrm{PT}x\right)\;,$$

we thus conclude that the action in Equation (12) is invariant under PT as long as the sources *s* and $\bar{s}$, which transform as functions,

(15) $$s\left(x\right)\overset{\mathrm{PT}}{\to }s^{*}\left(\mathrm{PT}x\right)\;,\;\bar{s}\left(x\right)\overset{\mathrm{PT}}{\to }{\bar{s}}^{*}\left(\mathrm{PT}x\right)\;,$$

are real.

With this in mind, the minimal ingredients needed to reproduce such a model in the gravity side are the following:

- A U(1) gauge symmetry dual to the global U(1) symmetry.
- A complex scalar field ϕ of mass $m^{2}=-2$ charged under the U(1) gauge symmetry dual to the operator O. The leading order ϕ in the expansion around the conformal boundary of AdS is identified with the source *s* (and correspondingly for $\bar{\phi }$).

Thus, the minimal gravity action chosen in [28] is given by

(16) $$S=\int d^{4}y\sqrt{-g}\left(R-2\Lambda -\frac{1}{4}F_{MN}F^{MN}-D_{M}\phi D^{M}\bar{\phi }-m^{2}\phi \bar{\phi }-\frac{v}{2}\phi^{2}{\bar{\phi }}^{2}\right)$$

where *A* is the gauge field of the U(1) symmetry, F=dA is the field strength tensor, and Λ=−3 is the cosmological constant. The coupling v=3 is added so that, at zero temperature, the dual QFT has an RG flow interpolating between two conformal field theories [32]. The action of the U(1) transformation is

(17) $$\phi \to e^{-iq\alpha }\phi \;,\;\bar{\phi }\to e^{iq\alpha }\bar{\phi }\;,\;A_{M}\to A_{M}-\partial_{M}\alpha \;,$$

and the covariant derivatives are defined as

(18) $$D_{M}\phi =\partial_{M}\phi -iqA_{M}\phi \;,\;D_{M}\bar{\phi }=\partial_{M}\bar{\phi }+iqA_{M}\bar{\phi }\;,$$

where *q* is the charge of O, which we set to unity unless stated otherwise.

Remarkably, from the point of view of the gravity theory, the non-Hermicity enters only through the boundary conditions on the conformal boundary (z=0), which explicitly read

(19) $$\begin{array}{cc} \left(z^{2}ds^{2}\right){|}_{z\to 0} & =\left[-dt^{2}+{\left(dx^{1}\right)}^{2}+{\left(dx^{2}\right)}^{2}+dz^{2}\right]\;,\\ \partial_{z}\phi \left(z=0\right)=s\;, & \;\partial_{z}\bar{\phi }\left(z=0\right)=\bar{s}\;,\;A_{\mu }\left(z=0\right)=0\;. \end{array}$$

It is worth noting that one can turn on sources for external gauge fields by modifying the boundary conditions of $A_{\mu }$. Concretely, if we take

(20) $$A_{\mu }\left(z=0\right)=a_{\mu },$$

this is dual to coupling the action in Equation (12) to an external gauge field $a_{\mu }$. In particular, the zero component of such gauge field $a_{0}$ can be interpreted as a chemical potential μ.

As reviewed in the introduction, PT-symmetric models can be mapped to Hermitian theories through a similarity transformation known as the Dyson map. Let us then discuss the implementation of the Dyson map in our holographic model as presented in [28] and further developed in [30,31]. To do so, we focus first on the QFT side. We start from a Hermitian theory with $s=\bar{s}=M$ and consider the following similarity transformation (Dyson map)

(21) $$O\to SO\;,\;\bar{O}\to S^{-1}\bar{O}\;,$$

where $S=e^{\beta (x)}$ is a complexified U(1) transformation. At leading order, this transformation yields the following non-Hermitian action:

(22) $$S=S_{\mathrm{CFT}}+\int d^{3}x\;\;\left(MSO+MS^{-1}\bar{O}+\frac{i}{q}\left(S^{-1}\partial_{\mu }S\right)J^{\mu }+O{\left(S^{-1}\partial_{\mu }S\right)}^{2}\right)\;,$$

where $J^{\mu }$ is the U(1) current and $iq^{-1}S^{-1}\partial_{\mu }S$ behaves as an external gauge field. The term proportional to the current follows trivially from considering the variation in $S_{\mathrm{CFT}}$ under an infinitesimal U(1) transformation

(23) $$\delta S_{\mathrm{CFT}}=\frac{i}{q}\int d^{3}x\;\;\left(S^{-1}\partial_{\mu }S\right)J^{\mu }+O{\left(S^{-1}\partial_{\mu }S\right)}^{2}\;.$$

Instead of considering the Dyson map in Equation (21), it is more convenient to reinterpret it by coupling the theory to an external gauge field $a_{\mu }$ and defining the Dyson map as the following external gauge transformation

(24) $$s\to sS^{-1}\;,\;\bar{s}\to \bar{s}S\;,\;a_{\mu }\to a_{\mu }+\frac{i}{q}S^{-1}\partial_{\mu }S\;,$$

which also reproduces the action in Equation (22) if we start from a Hermitian theory with $s=\bar{s}=M$ and $a_{\mu }=0$.

From the point of view of the gravitational theory, the Dyson map in Equation (21) is dual to a complexified gauge transformation in Equation (17) with $S=e^{-i\alpha }$ and α=iβ. Remarkably, this changes the boundary conditions in Equation (19) to

(25) $$\begin{array}{cc} \left(z^{2}ds^{2}\right){|}_{z\to 0} & =\left[-dt^{2}+{\left(dx^{1}\right)}^{2}+{\left(dx^{2}\right)}^{2}+dz^{2}\right]\;,\\ \partial_{z}\phi \left(z=0\right)=S^{-1}s\;, & \;\partial_{z}\bar{\phi }\left(z=0\right)=S\bar{s}\;,\;A_{\mu }\left(z=0\right)=\frac{i}{q}S^{-1}\partial_{\mu }S\;. \end{array}$$

where we have assumed that β does not depend on *z*. Hence, in the gravity side, we can also consider the alternative description of the Dyson map in Equation (24), where we interpret it as an external gauge transformation acting on the sources *s*, $\bar{s}$, and $a_{\mu }$.

Therefore, any theory that, under an external gauge transformation of the form in Equation (24), can be mapped to a Hermitian one, admits a Hermitian description capable of reproducing the same phenomenological results. We stress, however, that the Dyson map is not to be viewed as a gauge symmetry of the theory. Instead, theories connected by Dyson maps describe different physical settings that share equivalent phenomenology [30]. Thus, following [28], we choose to work with the sources *s*, $\bar{s}$ parametrized as

(26) $$s=\left(1-\eta \right)M\;,\;\bar{s}=\left(1+\eta \right)M\;,$$

where η encodes the non-Hermiticity and *M* is a dimensionful parameter.

Lastly, before moving on to discussing the phenomenology of this model, let us make an important technical clarification. When considering a non-Hermitian thermal QFT, it is non-trivial how to define expectation values of operators. As argued in [29], in this holographic setup, the prescription in Equation (9) implements the following definition of expectation values

(27) $$\left\langle O(t,x)\ldots \right\rangle =\mathrm{Tr}\{O\left(t,x\right)\ldots \;e^{-\beta H}\}/\mathrm{Tr}\{e^{-\beta H}\}\;,\;O\left(t,x\right)=e^{-iHt}O\left(x\right)e^{iHt}\;,$$

where the trace is defined as in Equation (4). (The time evolution of O(t) is such that static configurations exist). Hence, our construction naturally imposes biorthogonality. The thermal density matrix ϱ can be written as

(28) $$\varrho =\frac{e^{-\beta H}}{\mathrm{Tr}\{e^{-\beta H}\}}=\frac{\sum_{n}|n\rangle e^{-\beta E_{n}}\langle \tilde{n}|}{\mathrm{Tr}\{e^{-\beta H}\}}\;,$$

where $H|n\rangle =E_{n}|n\rangle$ and $H^{†}|\tilde{n}\rangle =E_{n}^{*}|\tilde{n}\rangle$. This is consistent with the standard definition of density matrix for pseudo-Hermitian quantum mechanics upon identifying $\langle \tilde{n}|=\langle n|\gamma$ with γ as the metric operator [8,12].

## 4. Phenomenology with Constant Sources

We begin now by reviewing some of the most relevant aspects of the model for constant sources. We focus on the phase diagram and the AC electric conductivity σ(ω) defined as

(29) $$\sigma \left(\omega \right)=\frac{\left\langle J_{1}\left(\omega \right)\right\rangle }{E_{1}\left(\omega \right)}\;,$$

where $\left\langle J_{1}\right\rangle$ and $E_{1}$ are the $x^{1}$-components of the expectation value of the current and of the electric field, respectively. Note that we have used the fact that the QFT is rotationally invariant in the $x^{1}-x^{2}$ plane to define a scalar conductivity as opposed to a tensorial one ($\sigma_{ij}=\delta_{ij}\sigma$). To compute this observable from the gravitational dual, one needs to consider linearized fluctuations of the gauge field $A_{1}$ (and of every other field that couples to it at the linear level). This is equivalent to computing the conductivity in linear response theory in the QFT. For a background with rotational invariance in the $x^{1}-x^{2}$ plane, one needs to consider fluctuations of the metric $g_{\mu \nu }$ and the gauge field $A_{\mu }$ of the form

(30) $$\delta A_{1}=\alpha \left(z\right)e^{-i\omega t}\;,\;\delta g_{01}=h_{01}\left(z\right)e^{-i\omega t}$$

where *z* is the holographic coordinate. Then, the conductivity reads

(31) $$\sigma \left(\omega \right)=\frac{\partial_{z}\alpha \left(z=0\right)}{i\omega \alpha (z=0)}\;.$$

Note that this is a direct application of the holographic dictionary, as $J_{1}$ is the operator dual to $\delta A_{1}$, and $E_{1}$ is minus the time derivative of the corresponding source. Hence, $\left\langle J_{1}\left(\omega \right)\right\rangle =\partial_{z}\alpha \left(z=0\right)$ and $E_{1}\left(\omega \right)=i\omega \alpha \left(z=0\right)$.

For the metric in this section, it is convenient to take the following ansatz in Poincaré coordinates

(32) $$ds^{2}=\frac{1}{z^{2}}\left(-ue^{-\chi }dt^{2}+{\left(dx^{1}\right)}^{2}+{\left(dx^{2}\right)}^{2}+\frac{dz^{2}}{u}\right)\;,$$

where *u* and χ are functions of the holographic coordinate *z*.

We divide this section in two subsections, discussing solutions with zero and non-zero chemical potential, respectively.

### 4.1. Phase Diagram and Conductivity at μ=0

**Figure 1:**
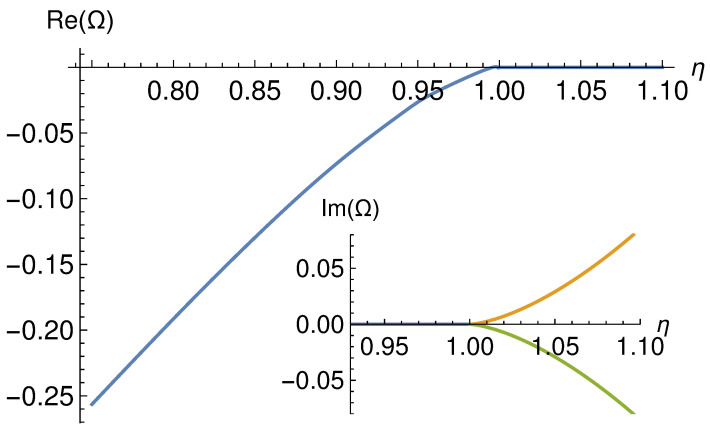
Free energy Ω for the model with constant sources at T=0 and μ=0. Note how, for |η|>1, the free energy becomes complex, thus signaling the spontaneous breaking of PT. The orange and green lines correspond to the two complex conjugate solutions existing for |η|>1. This figure is adapted from [28].

The phase diagram at zero chemical potential and zero temperature was first obtained in [28]. There, the authors showed that the system presents the following phases depending on the value of |η| (see Figure 1).

|η|<1: The QFT is in the PT-symmetric phase and the free energy is real. In the gravitational dual, the metric is real and the null energy condition (NEC) is satisfied.
|η|>1: The QFT is in the PT-broken phase. There are two branches of solutions whose free energies are complex conjugates of each other. In the gravitational dual, the metric is complex and the NEC is ill defined.
|η|=1: The QFT is at the exceptional point between the PT-symmetric and the PT-broken phases. In the gravitational dual, the metric and the NEC are satisfied. Remarkably, in this case, the scalar field decouples from the metric and does not backreact.

**Figure 2:**
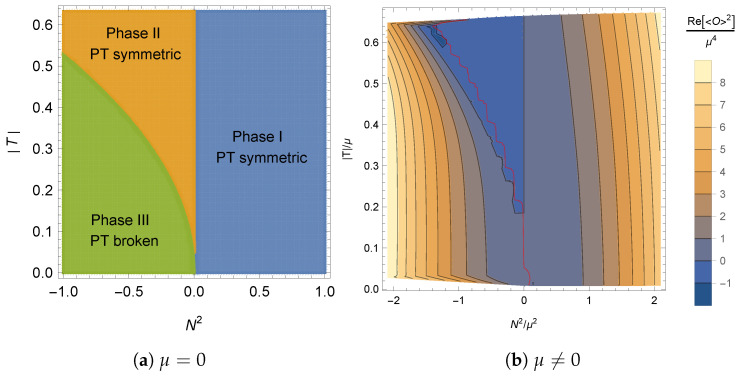
Phase diagram for the model with constant sources at zero chemical potential (**a**) and non-zero chemical potential (**b**). Here, $N^{2}=\left(1-\eta^{2}\right)M^{2}$. In (**b**), the color map represents the quantity $ℜ\left[{\left\langle O\right\rangle }^{2}\right]/\mu^{4}$. In (**b**), phase I is found in the region with $N^{2}/\mu^{2}>0$, and the red curve denotes the transition between phase II (right) and phase III (left). The superconducting phase appears for |T/μ|<0.02 and thus is not plotted. Both figures are taken from [29].

The phase diagram at non-zero temperature was constructed in [29], where the following phase structure was found (see Figure 2a).

**Phase I:**  In the region with |η|<1, the QFT is in a PT-symmetric phase and the free energy is real. In the gravitational dual, the metric is real and the null energy condition (NEC) is satisfied. The zero-temperature limit of this phase corresponds to the phase with |η|<1 above.
**Phase II:**  In the region with $1<|\eta |<\sqrt{1+\lambda_{c}{\left(T/M\right)}^{2}}$ with $\lambda_{c}\approx 3.6$, the QFT is in a PT-symmetric phase and the free energy is real. In the gravitational dual, the metric is real but the NEC is violated. This phase is linearly unstable. The zero-temperature limit of this phase corresponds to the exceptional point |η|=1 above.
**Phase III:**  In the region with $|\eta |>\sqrt{1+\lambda_{c}{\left(T/M\right)}^{2}}$, the QFT is in a PT-broken phase. There are two branches of solutions whose free energies are complex conjugates of each other. In the gravitational dual, the metric is complex, NEC is ill defined, and the temperature is complex. This phase is linearly unstable. The zero-temperature limit of this phase corresponds to the phase with |η|>1 above.

With regards to the conductivity, the authors of [29] found that all phases satisfy the Ferrel–Glover–Tinkham (FGT) sum rule

(33) $$\int_{-\infty }^{\infty }d\omega \left(Re\left[\sigma \left(\omega \right)\right]-1\right)=0\;.$$

This sum rule is derived from assumptions of causality, unitarity, and charge conservation; hence, it is quite remarkable that it still holds in phase III, where unitarity is violated due to PT being spontaneously broken.

**Figure 3:**
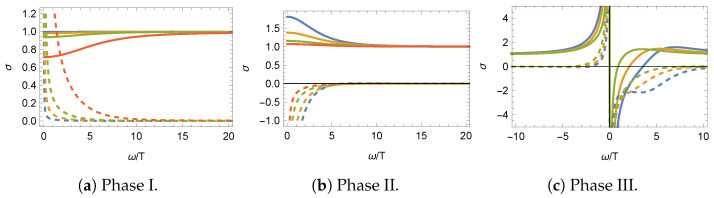
Conductivity σ in the three different phases of the model with constant sources for T≠0 and μ=0. The real (imaginary) part is denoted by solid (dashed) lines. The colors {blue, yellow, green, red} correspond to solutions with ⟨O⟩/(NT)={−1.6,−1.7,−2.0,−3.5} in (**a**) and to solutions with ⟨O⟩/(NT)={1.3,0,−0.9,−1.3} in (**b**). In (**c**), the colors {blue, yellow, green} correspond to solutions with $N^{2}/{\left|T\right|}^{2}=\left\{-17.0,-8.8,-4.6\right\}$. Here, $N^{2}=\left(1-\eta^{2}\right)M^{2}$. All figures are taken from [29].

The profile of the conductivity is shown in Figure 3 for the three different phases. As the QFT is conformal in the UV, the large frequency limit of the conductivity recovers the conformal result σ=1. At zero frequency, the real part of the conductivity displays a delta function typical of translational invariant systems. As the Krammers–Kronig relation is obeyed, the imaginary part features a 1/ω pole and indeed the low-frequency behavior of the conductivity is given by

(34) $$\sigma \left(\omega \right)=\rho \left(\pi \delta \left(\omega \right)+\frac{i}{\omega }\right)+\ldots \;,$$

where the ellipses denote terms regular in ω, and ρ is the total charge density (e.g., for a superfluid, ρ would be the sum of the superfluid charge density and the normal charge density). In phase I, the charge density satisfies ρ>0, and the conductivity obeys $\sigma {\left(\omega \right)}^{*}=\sigma \left(-\omega \right)$, as one would expect in a Hermitian theory. On the other hand, in phase II, the conductivity still fulfills $\sigma {\left(\omega \right)}^{*}=\sigma \left(-\omega \right)$, but the charge density is now negative ρ<0. Lastly, in phase III, the charge density becomes complex ρ∈C, and as PT is spontaneously broken, $\sigma {\left(\omega \right)}^{*}\neq \sigma \left(-\omega \right)$.

### 4.2. Phase Diagram and Conductivity at μ≠0

Solutions with μ≠0 were studied in [29]. In the presence of a chemical potential, the model in Equation (16) has a superconducting instability, where the scalar field ϕ acquires a non-trivial profile even in the absence of sources [33,34]. In the dual QFT, this corresponds to a phase transition from a normal fluid to a superfluid associated with the spontaneous symmetry breaking of the global U(1) symmetry. For |η|=1, the phase transition is second-order, while for |η|≠1, it is a crossover. (Properly speaking, the U(1) symmetry is not spontaneously broken, as it was already explicitly broken by the sources *s* and $\bar{s}$. This, in turn, implies that the superconductor phase transition is generically expected to be a crossover, as it is indeed observed).

The phase diagram can be seen in Figure 2b. Note that the superconducting phase only appears below |T/μ|≈0.02 and is not plotted in the figure. For temperatures above |T/μ|≈0.02, the phase structure matches the one found at zero chemical potential.

**Figure 4:**
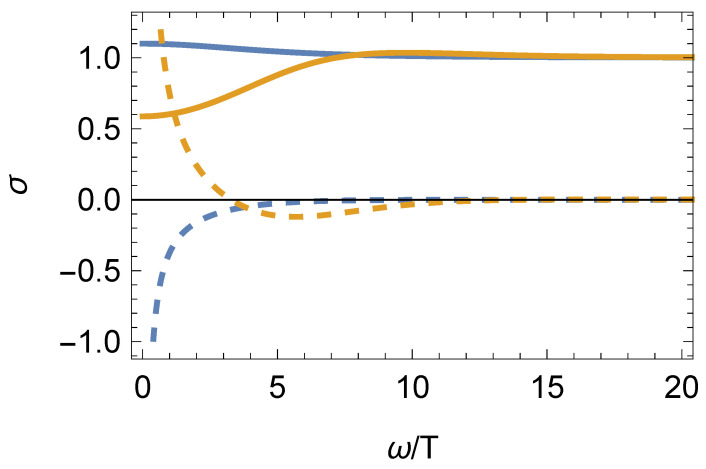
Conductivity σ in phase II for the model with constant sources at T≠0 and μ≠0. The real (imaginary) part is denoted by solid (dashed) lines. The blue (orange) line denotes a solution with $N^{2}/\mu^{2}=-6.2$ and T/μ = 2.4 ($N^{2}/\mu^{2}=-0.063$ and T/μ = 0.22). The change of sign in Imσ(ω→0) indicates a change in the sign of the charge density. Note how, at sufficiently small $|N^{2}/\mu^{2}|$, the charge density becomes positive as the normal component dominates over the negative contribution. Here, $N^{2}=\left(1-\eta^{2}\right)M^{2}$. This figure is taken from [29].

With regards to the conductivity, once again, the FGT sum rule holds for all phases. Moreover, the properties of σ(ω) are the same as those discussed in the previous section with only one minor difference in phase II. Now, as the fluid has non-zero chemical potential, the charge density obtains a positive contribution from the normal charge density, which can win against the negative contribution found in phase II at zero chemical potential. This behavior, illustrated in Figure 4, is only observed for sufficiently small values of the ratio $|\left(1-\eta^{2}\right)M^{2}/\mu^{2}|$.

## 5. Phenomenology with Spacetime-Dependent Sources

In this section, we will analyze holographic theories with spacetime-dependent non-Hermitian sources. We first consider quenches of those couplings and next study lattices where the non-Hermitian sources are periodic along a spatial direction.

The study of quantum field theories with spacetime-dependent sources is compelling because it reveals insights into how quantum fields respond to external conditions that vary across space and time, expanding our understanding of fundamental physics. Studying non-Hermitian PT-symmetric quantum field theories with spacetime-dependent sources is intriguing, as these theories challenge and expand the fundamental principles of quantum mechanics and field theory.

We are interested in non-Hermitian quenches, as they can be used to describe the rapid transition from a closed (Hermitian) system to an open (non-Hermitian) one. Non-Hermitian lattices instead describe physical setups where an influx/outflux of matter is distributed periodically along the system. They can also be useful to model a junction between a non-Hermitian and a Hermitian system.

### 5.1. Non-Hermitian Quenches

Quantum quench refers to a sudden change in the Hamiltonian of a quantum system at or around some specific time. More generally, we refer to a quench as a time dependence of the Hamiltonian localized in time around, say, t=0, and of a duration τ. Thus, for |t|≫τ, one is essentially dealing with a time-independent Hamiltonian. It is of highest interest to see how the initial ground state evolves in time and eventually settles down to a new ground state. In quantum field theory, such a problem is extremely difficult to address, especially in the strong coupling regime. It is rather remarkable that in gauge/gravity duality, the problem is much easier to deal with. Such holographic quantum quenches have led to important insights into questions of thermalization in strongly coupled systems (see, e.g., [35] for a review).

In the case of quantum mechanical systems with time-dependent pseudo-Hermitian Hamiltonians, two very valuable reviews are [6,8]. In gauge/gravity duality, a completely analog time dependence can be introduced in the sources at the boundary of the asymptotically AdS space, and this allows us to study non-Hermitian quantum quenches of strongly coupled holographic quantum field theories. In view of this, it is rather exciting to apply the method of holographic quantum quenches to the non-Hermitian model introduced in the previous section. This program has been carried out in [30]. We will now briefly review some of the most important results of this study. The holographic model in Equation (16) was solved in presence of time-dependent non-Hermitian sources. That is, we take a source configuration as in Equation (26), where now the non-Hermitian deformation is a time-dependent function η=η(t).

In order to study the time-dependent problem, it is helpful to use the following ansatz for the metric in terms of ingoing Eddington–Finkelstein coordinates

(35) $$\begin{array}{c} ds^{2}=-fdv^{2}-\frac{2}{z^{2}}e^{g}dzdv+h\left(dx^{2}+dy^{2}\right)\;, \end{array}$$

where *f*, *g*, and *h* are functions of the coordinates (v,z). We require the asymptotic boundary conditions

(36) $$\begin{array}{c} \lim_{z\to 0}\left(z^{2}f\right)=1\;\;\;,\;\;\;\lim_{z\to 0}\left(g\right)=0\;\;\;,\;\;\;\lim_{z\to 0}h=1\;, \end{array}$$

which fix the asymptotic AdS boundary at z=0. Naturally, we switch on the scalar fields ϕ and $\bar{\phi }$ as functions of (v,z) as well, and, additionally, one has to allow for a non-zero time component of the gauge field $A_{v}\left(v,z\right)$. Moreover, since we are interested in finite temperature solutions, we require our geometries to feature a non-degenerate planar apparent horizon where f=0. Its coordinate location can be set to z=1.

The equations of motion resulting from Equation (16) are now partial differential equations (PDEs). They were solved numerically in [30], where the time evolution of the following operators was monitored: the scalar operators O and $\bar{O}$ dual to the scalar fields ϕ and $\bar{\phi }$; the energy density $T_{vv}$ and pressure $T_{xx}=T_{yy}$ dual to the metric components $g_{vv}$ and $g_{xx}$, $g_{yy}$; and the charge density $J^{v}$ dual to the gauge field $A_{v}$. It is important to note that we do not source the gauge field explicitly, and due to the non-Hermitian nature of the boundary conditions, the gauge field turns out to be purely imaginary. Full details on the expression of these operators in terms of the AdS fields and on the numerical procedures can be found in [30].

The non-Hermitian (holographic) quantum quench is implemented by the following choice of profile for η

(37) $$\begin{array}{c} \eta \left(v\right)=\eta_{i}+\frac{\eta_{f}-\eta_{i}}{2}\left[1+\tanh\left(\frac{v-v_{m}}{\tau }\right)\right]\;. \end{array}$$

**Figure 5:**
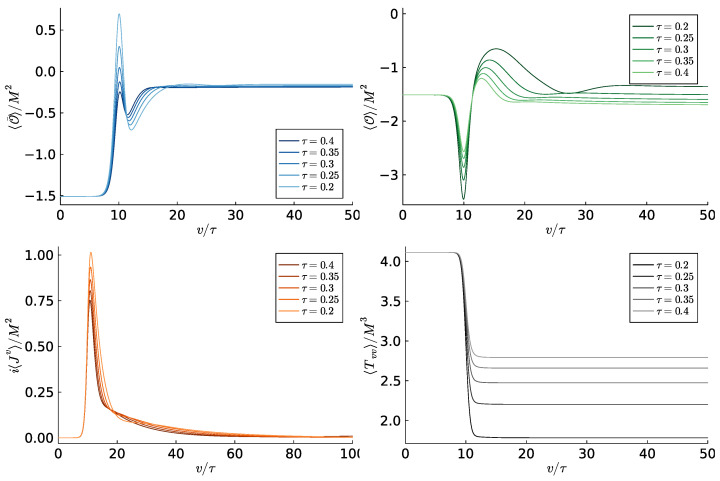
Expectation values of scalar operators, (imaginary) charge density and energy density for a quench described by the profile in Equation (37) for several values of τ, which interpolates between the Hermitian point $\eta_{i}=0$ and a final value of $\eta_{f}=0.8\;$ (Adapted from [30]).

The initial value $\eta_{i}$ is chosen to be the Hermitian theory $\eta_{i}=0$, whereas the endpoint is taken to be $\eta_{f}=0.8$. We remind the reader that the exceptional point at the phase transition towards the PT-broken phase is η=1. The results of such quantum quenches with $v_{m}=10\tau$ for different values of τ are shown in Figure 5.

**Figure 6:**
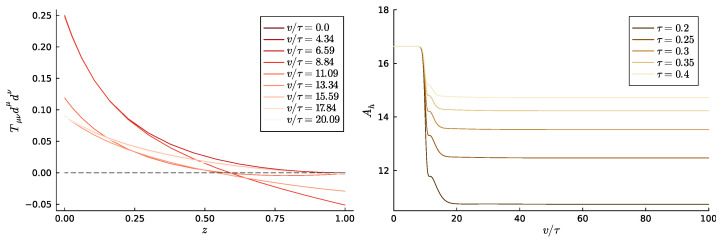
**Left**: The null energy condition $T_{\mu \nu }d^{\mu }d^{\nu }\geq 0$ for the particular simulation of Figure 5 with τ=0.4 at different stages v/τ of the evolution. It is clearly violated, especially at the horizon z=1. **Right**: We display the size of the apparent horizon for the simulations of Figure 5. All of them show a shrinking horizon, which is tightly related to violating the NEC.

The most interesting feature is that the energy of the dual field theory decreases. Since the start and end points are black branes, this means that the temperature of the black brane is actually lowered by the non-Hermitian quench. In fact, the apparent horizon shrinks during the time evolution. This shrinking of the horizon is not in contradiction to the second law of black hole thermodynamics, which states that the area of the horizon has to grow in any given process, provided the null energy condition (NEC) is satisfied. Indeed, during the non-Hermitian quench, the NEC is violated. It suffices to take the null vector tangent to infalling null geodesics $d=\partial_{v}-\frac{1}{2}z^{2}fe^{-g}\partial_{z}$. Then, $T_{\mu \nu }d^{\mu }d^{\nu }$, where $T_{\mu \nu }$ is the bulk energy momentum tensor, should be positive if the NEC was to hold. The violation of the NEC can be clearly seen in Figure 6.

We note that [30] also investigated quenches that end in the exceptional point. There, it was found that the endpoint $\eta_{f}=1$ settled down to new equilibrium configurations with non-vanishing charge density. Until now, we have considered the scenario where η corresponds to quenches in which a non-Hermitian operator is time-dependent. Instead, one can study a setup where the non-Hermitian coupling, $s-\bar{s}$, is kept constant while the Hermitian one, $s+\bar{s}$, is quenched. Interestingly, in this case, the NEC is still violated in the bulk of the spacetime but not at the horizon. Consequently, for such quenches, the black brane horizon grows!

Finally, we could instead consider a non-Hermitian time-dependent configuration of sources equivalent via the Dyson map in Equation (24) to a Hermitian quench. It is clear from the form of Equation (24) that one needs to switch on a purely imaginary source for the gauge field $A_{v}$ to have such a non-Hermitian configuration. Indeed, in [30], it was checked that the time evolution of the holographic model satisfying the asymptotic boundary conditions

(38) $$\partial_{z}\phi \left(z=0,v\right)=\left(1-\eta \right)M\;,\;\partial_{z}\bar{\phi }\left(z=0,v\right)=\left(1+\eta \right)M\;,\;a_{v}\left(z=0,v\right)=\frac{i}{q}\frac{\partial_{v}\eta }{1-\eta^{2}}\;,$$

results in the same dynamics as that obtained from imposing

(39) $$\partial_{z}\phi \left(z=0,v\right)=\sqrt{1-\eta^{2}}M\;,\;\partial_{z}\bar{\phi }\left(z=0,v\right)=\sqrt{1-\eta^{2}}M\;,\;a_{v}\left(z=0,v\right)=0\;,$$

which is the source configuration resulting from applying the map in Equations (24) to (38), with $S^{-1}=\sqrt{\left(1+\eta )/(1-\eta \right)}$.

### 5.2. Non-Hermitian Lattices

In this subsection, we review the results of [31] and consider setups where the non-Hermitian deformation of the holographic theory is inhomogeneous; namely, we make η a function of a spatial coordinate. In particular, we take the sources *s* and $\bar{s}$ to be functions of the coordinate $x^{1}$. In [31], this allowed the authors to build a *non-Hermitian lattice* and a *non-Hermitian junction.* The former is a lattice, where, in each site, there is an inhomogeneous (space-dependent) inflow/outflow of matter; and the latter is a Hermitian/non-Hermitian/Hermitian junction which could describe a lattice system, where, in one site, there is an impurity causing the inflow/outflow. For the sake of conciseness, here we focus only on the lattice case. In [31], this setup was studied in more detail due to its greater numerical simplicity. Similar conclusions are expected to hold for the junction.

The lattice is characterized by the following inhomogeneous non-Hermitian parameter

(40) $$\eta \left(x^{1}\right)=a\cos\left(\frac{2\pi }{L}x^{1}\right)\;,$$

and a vanishing external gauge field ($a_{\mu }=0$). (In [31], the authors also considered non-vanishing $a_{1}=iq^{-1}\partial_{1}\eta /\left(1-\eta^{2}\right)$. This allowed them to explicitly check that, as pointed out in [30], that non-Hermitian model is a Dyson map of the Hermitian theory). Due to the conformal symmetry of the UV, the non-Hermitian lattices are thus characterized by three parameters, the temperature T/M, the cell length LM, and the amplitude *a*.

The dual geometry is going to be inhomogeneous along the $x^{1}$ direction and can be solved by means of the following metric ansatz:

(41) $$\begin{array}{cc} ds^{2}=\frac{1}{z^{2}} & \left[-\left(1-z^{3}\right)h_{1}dt^{2}+h_{3}{\left(dx^{1}+h_{5}\;dz\right)}^{2}+h_{4}{\left(dx^{2}\right)}^{2}+\frac{h_{2}}{1-z^{3}}dz^{2}\right]\;, \end{array}$$

where $h_{1}$, …, $h_{5}$ are functions of (z,x). Note that this ansatz has residual gauge symmetries that have not been removed. Thus, the PDEs resulting from the equations of motion are solved numerically using the DeTurck trick [31].

**Figure 7:**
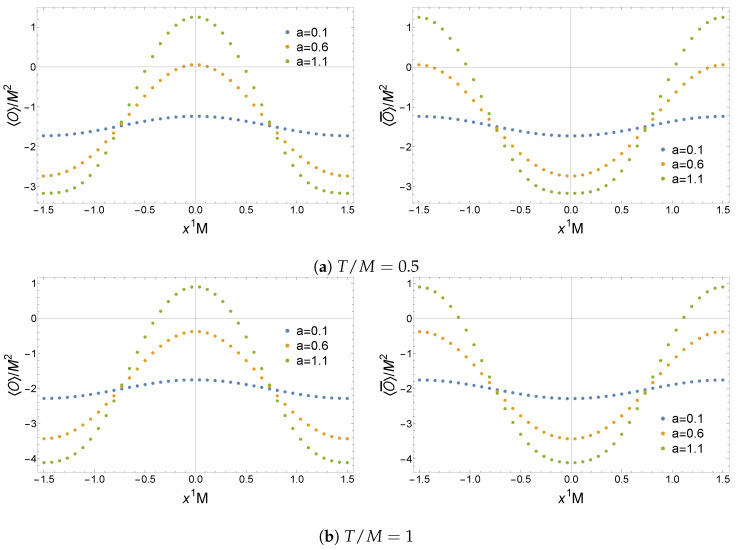
Expectation values of the operators ⟨O⟩ and $\left\langle \bar{O}\right\rangle$ in a non-Hermitian lattice with LM=3. Figure taken from [31].

**Figure 8:**
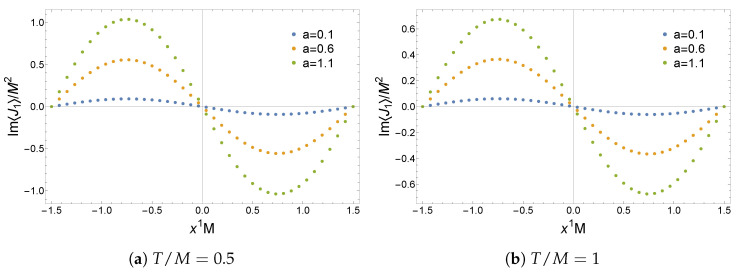
Imaginary part of the expectation value of the current $\left\langle J_{1}\right\rangle$ in a non-Hermitian lattice with LM=3. The real part is zero for any {a,T/M}. Figure taken from [31].

**Figure 9:**
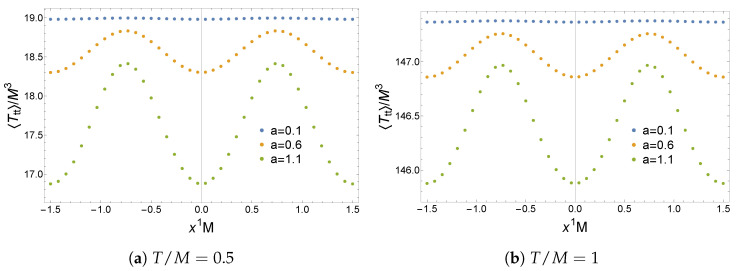
Expectation value of the energy density $\left\langle T_{tt}\right\rangle$ in a non-Hermitian lattice with LM=3. Figure taken from [31].

The expectation values of the operators ⟨O⟩ and $\left\langle \bar{O}\right\rangle$, the current $\left\langle J_{1}\right\rangle$, and the energy density $\left\langle T_{tt}\right\rangle$ for the non-Hermitian lattice are plotted in Figure 7, Figure 8 and Figure 9. Remarkably, these solutions have a real geometry and present a purely imaginary U(1) current previously absent in the homogeneous setup. Most notably, this current is an odd function of $x^{1}$ and thus spontaneously breaks PT

(42) $$\left\langle J_{1}\left(x\right)\right\rangle \overset{\mathrm{PT}}{\to }\left\langle J_{1}\left(\mathrm{PT}x\right)\right\rangle =-\left\langle J_{1}\left(x\right)\right\rangle \neq \left\langle J_{1}\left(x\right)\right\rangle \;,$$

indicating that this model cannot be mapped to a purely Hermitian theory via the Dyson map in Equation (24). Hence, inhomogeneities allow one to construct PT-breaking solutions that do not have complex geometries. This is a generic feature of these solutions that holds for a≠0. For |a|>1 sufficiently large at any fixed T/M and LM, one finds that real solutions no longer exist. This resembles the observation of [28] for the homogeneous solutions of phase II. Hence, it seems likely that complex solutions will dominate in some regions of the parameter space where |a| is large.

**Figure 10:**
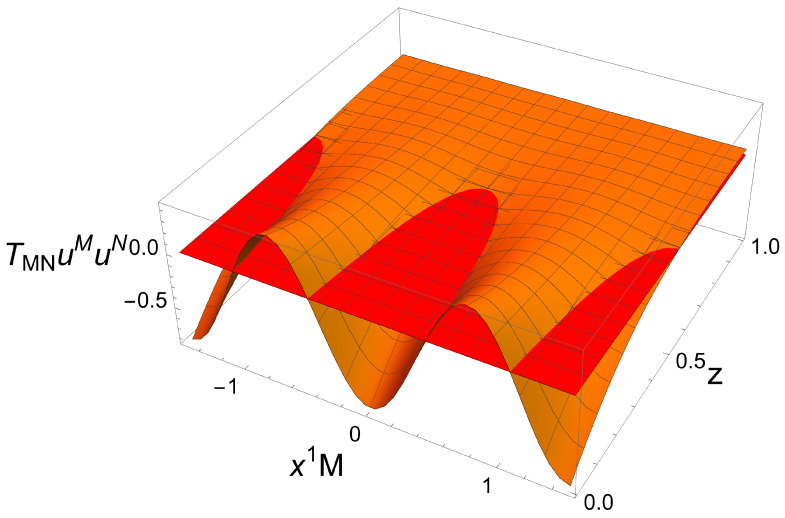
Violation of the NEC for a stable non-Hermitian lattice with T/M=0.5, LM=3, and a=1.7. The orange surface denotes $T_{MN}u^{M}u^{N}$ and the red one is the surface $T_{MN}u^{M}u^{N}=0$ for reference. Here, *u* is an infalling null geodesic. Figure taken from [31].

It is also worth mentioning that, unlike the homogeneous setup, stable PT-breaking solutions were found even for setups with |a|>1 locally satisfying |η|>1 (which was the onset of instability for the homogeneous case). However, the solutions with |a|>1 do violate the NEC close to the AdS boundary (see Figure 10), although it is preserved near the event horizon.

For solutions with |a|>1, a remarkable implication of the violation of the NEC near the AdS boundary is that the standard *a*-function [36,37,38] locally increases towards the IR in the region where $|\eta (x^{1})|>1$. The *a*-function encodes the number of degrees of freedom along the renormalization group flow of the dual QFT. Hence, these solutions are potentially problematic as the number of degrees of freedom seemingly increases as one integrates out from the UV to the IR. However, the failure of the standard a-function to be monotonically decreasing alone is not enough to conclusively prove this. To ensure that indeed the number of degrees of freedom increases, one would need to prove the non-existence of a monotonically decreasing function behaving as the central charge for z→0.

**Figure 11:**
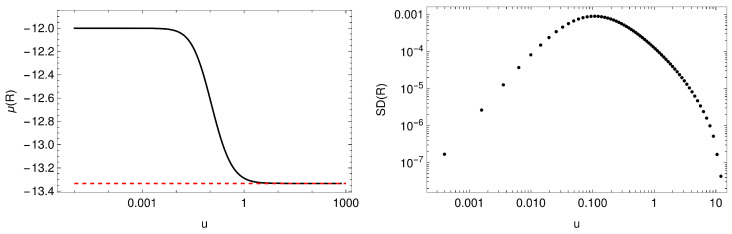
**Left**: spatial average of the Ricci scalar *R* at zero temperature. The red dashed line shows the value R=−12−4v corresponding to the IR fixed point in Equation (44). **Right**: standard deviation of *R* at zero temperature. The radial coordinate *u* is defined as u=z/(1−z).

**Figure 12:**
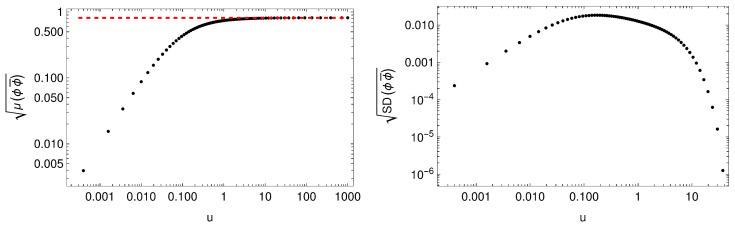
**Left**: square root of the spatial average of $\phi \bar{\phi }$ at zero temperature. The red dashed line depicts the value $\sqrt{2/v}$ corresponding to the IR fixed point in Equation (44). **Right**: square root of the standard deviation of $\phi \bar{\phi }$ at zero temperature. As above, u=z/(1−z).

In [31], the authors also studied the zero-temperature limit of the non-Hermitian lattice for |a|<1. They found that the zero-temperature IR geometry corresponds to that of a Hermitian conformal fixed point [32]

(43) $$\begin{array}{cc} ds^{2}= & \frac{3v}{\left(1+3v\right)z^{2}}\left(-dt^{2}+{\left(dx^{1}\right)}^{2}+{\left(dx^{2}\right)}^{2}+dz^{2}\right)\;,\\ \; & \phi =\bar{\phi }=\sqrt{\frac{2}{v}}\;,\;A_{1}=0\;, \end{array}$$

rotated under a complexified U(1). This is illustrated in Figure 11 and Figure 12, where we plot the standard deviation and the mean value of the Ricci scalar *R* and the U(1) invariant $\phi \bar{\phi }$, observing that they tend to the values at the conformal fixed point R=−12−4/v and $\sqrt{\phi \bar{\phi }}=\sqrt{2/v}$. (Notice that to find the zero-temperature solutions one needs to modify the ansatz in Equation (41) as indicated in [31]. Moreover, the charge is set to q=2 since, as noted in [39], this choice ensures the existence of a conformal IR fixed point for our choice of parameters).

In more detail, the authors found that the solution near the IR had the following form

(44) $$\begin{array}{cc} ds^{2} & =\frac{3v}{\left(1+3v\right)z^{2}}\left(-dt^{2}+{\left(dx^{1}\right)}^{2}+{\left(dx^{2}\right)}^{2}+dz^{2}\right)\;,\\ \phi & =S\sqrt{\frac{2}{v}}\;,\;\bar{\phi }=S^{-1}\sqrt{\frac{2}{v}}\;,\;A_{1}=-\frac{i´}{q}S^{-1}\partial_{1}S\;, \end{array}$$

with *S* being an $x^{1}$-dependent complexified U(1) rotation given by

(45) $$S=\sqrt{\frac{1-\tilde{\eta }}{1+\tilde{\eta }}}\;,$$

where numerically it was observed that the function $\tilde{\eta }$ satisfied

(46) $$\frac{\tilde{\eta }}{\sqrt{1-{\tilde{\eta }}^{2}}}=\eta \;.$$

In conclusion, the IR geometry restores PT, as a Dyson map of the form in Equation (24) connects it to the Hermitian fixed point in Equation (43), which is PT-unbroken. Hence, the non-Hermitian lattices flow from a PT-broken UV to a PT-unbroken theory in the IR. Quite remarkably, the IR PT symmetry restoration observed in this strongly coupled model was also found in the perturbative regime in [21,22]. There, the authors found that small non-Hermitian deformations to the mass matrix of a doublet model lead to a theory interpolating between a PT-broken, non-unitary UV, and a PT-symmetric, unitary IR. This suggests that this phenomenon might be a generic property of inhomogeneous non-Hermitian systems, although further studies are needed to determine if this is indeed the case.

## 6. Conclusions

Non-Hermitian QFTs are very interesting due to their novel phenomenology absent in their Hermitian counterparts. They allow us to describe open systems and by tuning the matter flow, we can achieve stationary solutions where unitary evolution can be realized. This is directly related to the existence of an unbroken antilinear $Z_{2}$-symmetry, which is typically taken to be PT, which guarantees that the energy spectrum is real.

Much of the study of non-Hermitian QFTs has been restricted to the perturbative regime for its simplicity. However, in order to deepen our understanding of these theories, it is interesting to extend such studies to the strong coupling regime. In this context, holographic non-Hermitian QFTs provide an interesting path as they offer a controlled non-perturbative setup.

In this work, we have collected the key developments in the study/field of holographic non-Hermitian field theories. We have reviewed the construction of a gravity dual to a CFT where a non-Hermitian deformation is introduced through the sources of a complex scalar operator [28]. The model is PT-symmetric and features a rich phase diagram with phases where PT is spontaneously broken and phases where it is not [29]. We have shown how the Dyson map, which maps PT-symmetric phases to a Hermitian description, is implemented in the gravity dual. We next considered configurations where the non-Hermitian deformation is spacetime-dependent [30]. We distinguished two cases: quenches [30] and lattices [31]. In the former, the non-Hermitian deformation is time-dependent, while in the latter, it is space-dependent. For quenches from a Hermitian theory to a non-Hermitian theory, it was observed that the apparent horizon of the black brane shrinks, indicating that the dual field theory decreases its entropy. On the other hand, for the lattices, an spontaneous breaking of PT with real geometries was observed. Furthermore, this symmetry was restored in the IR, indicating that the model presents a PT-restoring RG flow.

## 7. Future Directions

We conclude this review with a list of possible future research directions in the context of non-Hermitian holography:

- **Construct holographic models with non-Hermitian SU(2) sources**. The existing literature on non-Hermitian models in holography has focused on the case where the non-Hermiticity arises from the sources of a scalar operator O charged under U(1). It would be interesting to generalize these ideas to models with non-Hermitian sources for operators charged under SU(2). In the context of spacetime-dependent sources, one could think of this setup as a strongly coupled generalization of the models of [21,22].
- **Fermi surfaces in non-Hermitian holography**. In [40], non-Hermitian Dirac cones were studied and experimentally realized in an optical setting. It would be interesting to study the effect of non-Hermiticity on the Fermi surface of the holographic model with constant sources to compare with those results. Fermi surfaces correspond to the poles of fermionic correlators, which are dual to quasinormal modes of probe fermions in the bulk.
- **Non-hermitian cooling**. We have highlighted the interesting phenomenon that non-Hermitian holographic quantum quenches can violate the null energy condition at the horizon of an asymptotically AdS black hole. This makes the (apparent) horizon shrink and leads effectively to a lowering of the Hawking temperature. To our knowledge, this effect of non-Hermitian cooling has not been investigated yet at weak coupling. It would be interesting to study it in non-holographic models. Since PT-symmetric non-Hermitian quantum systems can be realized in experiments, it is also tempting to contemplate if such a non-Hermitian quantum cooling could be observed in the laboratory.
- **Phase diagram of non-Hermitian lattices**. In [29], the full phase diagram was constructed for the model with constant sources. It would be interesting to generalize such phase diagrams to the case of inhomogeneous sources. In particular, one could study the existence of the solutions with complex geometries and whether such solutions are thermodynamically preferred.
- **Universal nature of IR PT restoration in inhomogeneous models**. Perturbatively, it was observed in [21] that PT symmetry is restored in inhomogeneous non-Hermitian models with a PT-broken UV. This same phenomenon was also observed in the non-Hermitian lattice of [31] reviewed here. It would be interesting to explore whether this IR PT symmetry restoration is a generic property of inhomogeneous non-Hermitian field theories.
- **Non-Hermitian deformations around quantum critical points**. In [41], it was observed that a non-Hermitian lattice can qualitatively modify the dynamics in the vicinity of a quantum critical point. Via holography, one can explore these effects on strongly coupled systems.
- **Gravity models with a complexified gauge theory**. As the Dyson map corresponds to a complexified U(1) transformation, it would be interesting to consider a model where, instead of considering a U(1) gauge symmetry in the gravity side, we would consider a complex U(1) gauge symmetry. This approach could perhaps offer a more unified view of the relation between Dyson maps and complexified U(1) transformations.
